# Improvement of macrolactins production by the genetic adaptation of *Bacillus siamensis* A72 to saline stress via adaptive laboratory evolution

**DOI:** 10.1186/s12934-022-01871-9

**Published:** 2022-07-19

**Authors:** Yuman Gan, Meng Bai, Xiao Lin, Kai Liu, Bingyao Huang, Xiaodong Jiang, Yonghong Liu, Chenghai Gao

**Affiliations:** Institute of Marine Drugs, Guangxi University of Chinese Medicine, Guangxi, 530001 People’s Republic of China

**Keywords:** Macrolactins, Adaptive laboratory evolution, Saline tolerance, Amino acid metabolism, Feedback inhibition

## Abstract

**Background:**

Macrolactins, a type of macrolide antibiotic, are toxic to the producer strains. As such, its level is usually maintained below the lethal concentration during the fermentation process. To improve the production of macrolactins, we applied adaptive laboratory evolution technology to engineer a saline-resistant mutant strain. The hypothesis that strains with saline resistance show improved macrolactins production was investigated.

**Results:**

Using saline stress as a selective pressure, we engineered a mutant strain with saline resistance coupled with enhanced macrolactins production within 60 days using a self-made device. As compared with the parental strain, the evolved strain produced macrolactins with 11.93% improvement in non-saline stress fermentation medium containing 50 g/L glucose, when the glucose concentration increased to 70 g/L, the evolved strain produced macrolactins with 71.04% improvement. RNA sequencing and metabolomics results revealed that amino acid metabolism was involved in the production of macrolactins in the evolved strain. Furthermore, genome sequencing of the evolved strain revealed a candidate mutation, *hisD*^D41Y^, that was causal for the improved MLNs production, it was 3.42 times higher than the control in the overexpression *hisD*^D41Y^ strain. Results revealed that saline resistance protected the producer strain from feedback inhibition of end-product (macrolide antibiotic), resulting in enhanced MLNs production.

**Conclusions:**

In the present work, we successfully engineered a mutant strain with enhanced macrolactins production by adaptive laboratory evolution using saline stress as a selective pressure. Based on physiological, transcriptomic and genetic analysis, amino acid metabolism was found to benefit macrolactins production improvement. Our strategy might be applicable to improve the production of other kinds of macrolide antibiotics and other toxic compounds. The identification of the *hisD* mutation will allow for the deduction of metabolic engineering strategies in future research.

**Supplementary Information:**

The online version contains supplementary material available at 10.1186/s12934-022-01871-9.

## Introduction

Macrolactins (MLNs) are a type of 24-membered ring macrolides that are generally biosynthesized by type I polyketide synthase (PKS), and more than 32 monomers have been identified [1]. MLNs have been shown to have antibacterial activities against *Burkholderia cepacia* [2] and the agricultural pathogens *Alternaria* and *Pyricularia oryzae* [3], as well as antiviral activity against SARS-CoV-2 [4]. Obviously, MLNs have potential agricultural applications in controlling crop pests and disease treatment. However, the extreme scarcity of MLNs production has precluded further investigation. Due to this limitation, efforts are required to improve the production of MLNs.

The optimization of culture conditions had been shown to be an efficient strategy in increasing the yield of fermentation products [5], including MLN A [6], vitamin B12 [7], and angucycline [8]. However, this strategy is typically tedious, and it does not change the genetics of strains. To enhance the polyketide production in host strains, metabolic engineering approaches have been applied successfully, e.g., overexpression of rate-limiting enzymes [9] or PKS modules [10]. However, there are other obstacles to polyketide production improvement. Many secondary metabolites (e.g., antibiotics and pigments) are toxic to the producer strains [11], resulting in enzyme inactivation or even cell death. For example, daunorubicin, a member of the type II polyketide family, exerts feedback inhibition by DNA intercalation, leading to the down regulation of PKS genes [12]. Since producer strains are subjected to feedback inhibition, the production of polyketide or antibiotic is usually maintained below the lethal concentration during the fermentation process [13]. Thus, to improve the production of these compounds, it is important to remove the feedback inhibition. Various studies regarding the removal of feedback inhibition by end-products have been performed. The ABC system conferred in *Streptomyces peucetius* for self-resistance functions as an ATP-driven pump for the efflux of daunorubicin [14]. Although rational metabolic engineering approaches have been demonstrated to remove feedback inhibition, leading to improvements in the product yield or other characteristics of the strains [15, 16], these approaches are difficult to apply to organisms with partially annotated genetic material, or lacking genetic manipulation.

Adaptive laboratory evolution (ALE) is a powerful technique for improving or creating the microbial phenotypes of interest [17–19]. Unlike rational metabolic engineering, ALE does not require researchers to gain a better understanding of the genetic information of the desired phenotype. Using ALE technology, researchers have obtained several stress-resistant mutants, including ferulic acid resistance [20], methanol resistance [21], anthranilate resistance [22], isobutyl acetate resistance [23], and antibiotic overproduction mutant [24]. Based on the transcriptomic and/or genomic analysis results of the evolved organism, molecular mechanisms regarding the interesting phenotype are studied. Mo et al. revealed that secretory pathways transport proteins and lipids onto the plasma membrane to repair cell wall/membrane damaged by stress [25]. Gupta et al. found that genes involving amino acid metabolism, sugar uptake, membrane transport, and cell wall synthesis were maximally upregulated in isobutanol resistance. Among these, pathways including membrane transport and cell wall synthesis might provide mechanical strength for the strain to withstand extreme pressure [26].

ABC transport system are widely distributed in almost all species, and it may serve as import or export machineries of compounds ranging from inorganic ions, amino acids, polysaccharides and antibiotics to peptides [27, 28]. Feng et al. reported that ABC system exhibited dual functions as an antibiotic efflux pump and a NaCl anti-porter [29]. Combined with the previous report that ABC system participated in removing feedback inhibition of polyketide [14], it is reasonable for us to deduce that removing the feedback inhibition of polyketide shared the same molecular mechanism with NaCl-tolerance phenotype; namely, strains with NaCl-tolerance would show resistance to their toxic end-product polyketide, which would allow for increased production of these toxic end-products. Thus, in this study, ALE was applied to improve the saline tolerance of *Bacillus siamensis* A72 (A72) to remove feedback inhibition from MLNs production, thereby improving the yield of MLNs. Successive cultivation in the presence of saline stress, a saline-resistant mutant with enhanced MLNs production was obtained. To gain insights into the underlying molecular mechanism, we characterized the omics properties of the saline-resistant mutant. The results indicated that amino acid metabolism is a critical factor that attributed to the improvement in MLNs production and biological activity of the saline-resistant mutant strain.

## Results

### Using saline as screening pressure to assist strain evolution

In this study, ALE was applied to develop a mutant strain for improving MLNs production. A72 showing excellent MLNs metabolic performance was selected as the parental strain for ALE [30]. Prior to initiating a random mutant experiment, i.e., ALE, an appropriate screening strategy is a contributing factor that determines whether the whole experiment is a success or not. Based on the above-mentioned deduction, that improving saline resistance of strains might release the feedback inhibition of toxic polyketide, and eventually improving the polyketide production. Thus, in this study, saline stress was used as the screening pressure for improving the MLNs production of A72. To determine the starting saline concentration for the selection of saline-resistant strains, saline ranging from 3 to 10% (w/v) was directly added to the medium, and the growth kinetics was measured. The OD600nm value of A72 decreased by approximately 50% at 6% (w/v) saline concentration (Additional file 1: Fig. S1), and this concentration was selected as the starting concentration for ALE.

**Fig. 1:**
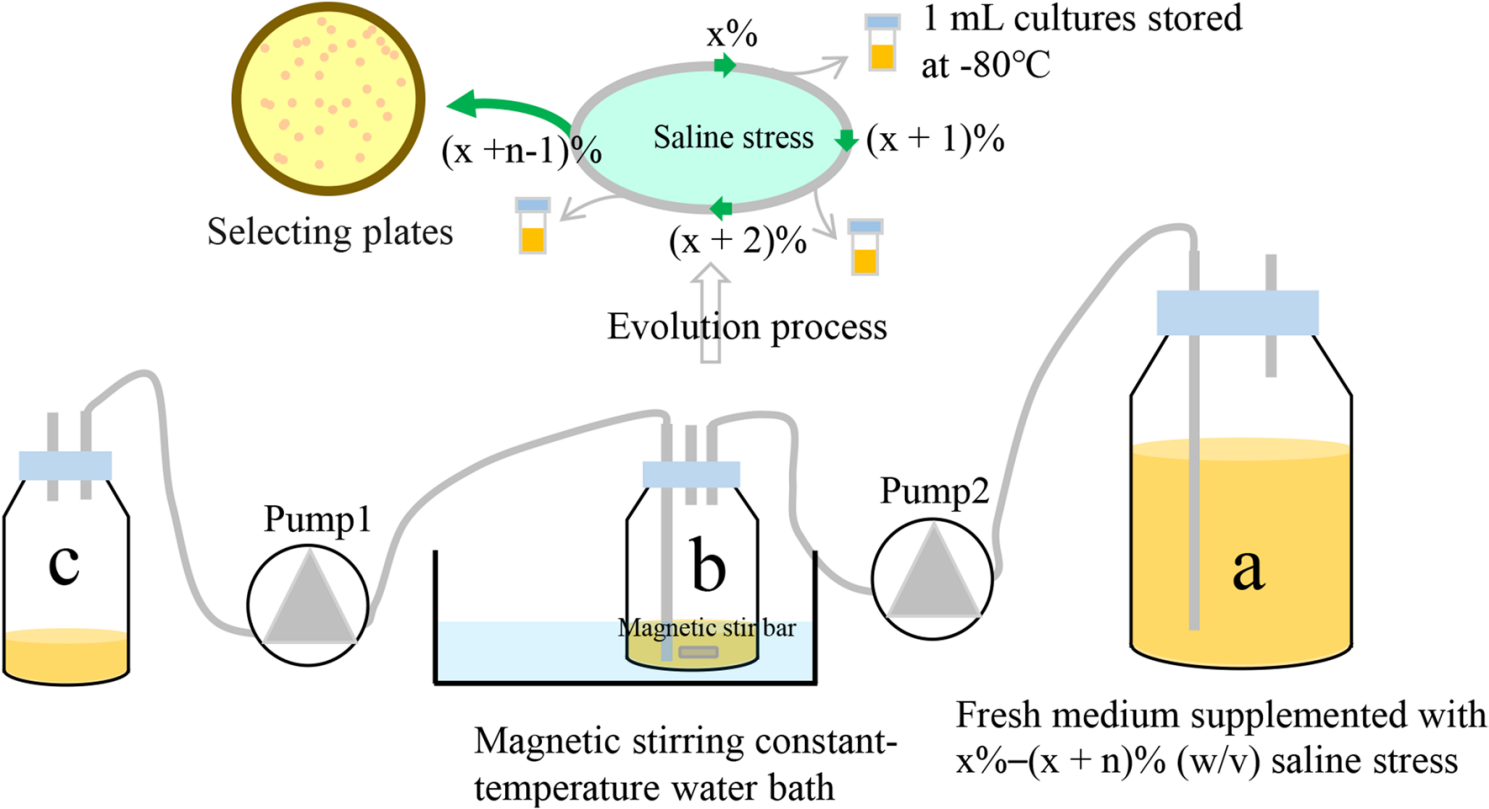
Schematic representation of the self-made device used for the evolution of *Bacillus siamensis* A72. The “**a**” container used for supplying the fresh medium for the evolution experiment. The “b” container used for culturing the strains at different saline concentrations. The “**c**” container used for collecting discarded evolution cultures. “x%” represents initial saline concentration. “n” represents transfer times. Cells were inoculated into “**b**” container with 30 mL medium containing x% (w/v) saline, to an initial OD600nm of approximately 0.3, cultured at 30 °C, and dissolved in oxygen by magnetic bar stirring. When the OD600nm value reached 5.0–8.0, approximately 25 mL of the culture was pumped out from the “**b**” container to “**c**” container, and 25 mL of fresh medium from the “**a**” container was pumped into “**b**” container. For each passage, 1.0 mL of the culture was stored in 30% (v/v) glycerol at −80 °C for subsequent experiments. When the medium (1000 mL) in “a” container was exhausted, refilled sterile fresh medium with the saline concentration increased by 1%. When the cells could no longer grow at a specific saline concentration in ISP-50-S medium, the ALE experiment was terminated, “n-1” passage cultures were used for selecting desire mutants

Since the ALE experiment is usually time-consuming, we assembled a simple device assisting A72 evolution to reduce labor intensity. The device consisted of three blue-cap bottles, two pumps, and a water bath (Fig. 1). The “a” container was filled with 1 L of ISP-50 medium supplemented with a specific saline concentration, which supplied the fresh medium for the evolution experiment. The “b” container was set at 30 °C and was used for culturing the strains at different saline concentrations. The “c” container was used for collecting discarded evolution cultures. Further details regarding the experimental setup of this device have been described in the “Materials and methods” section.

### Selecting the robust evolved strains

**Fig. 2:**
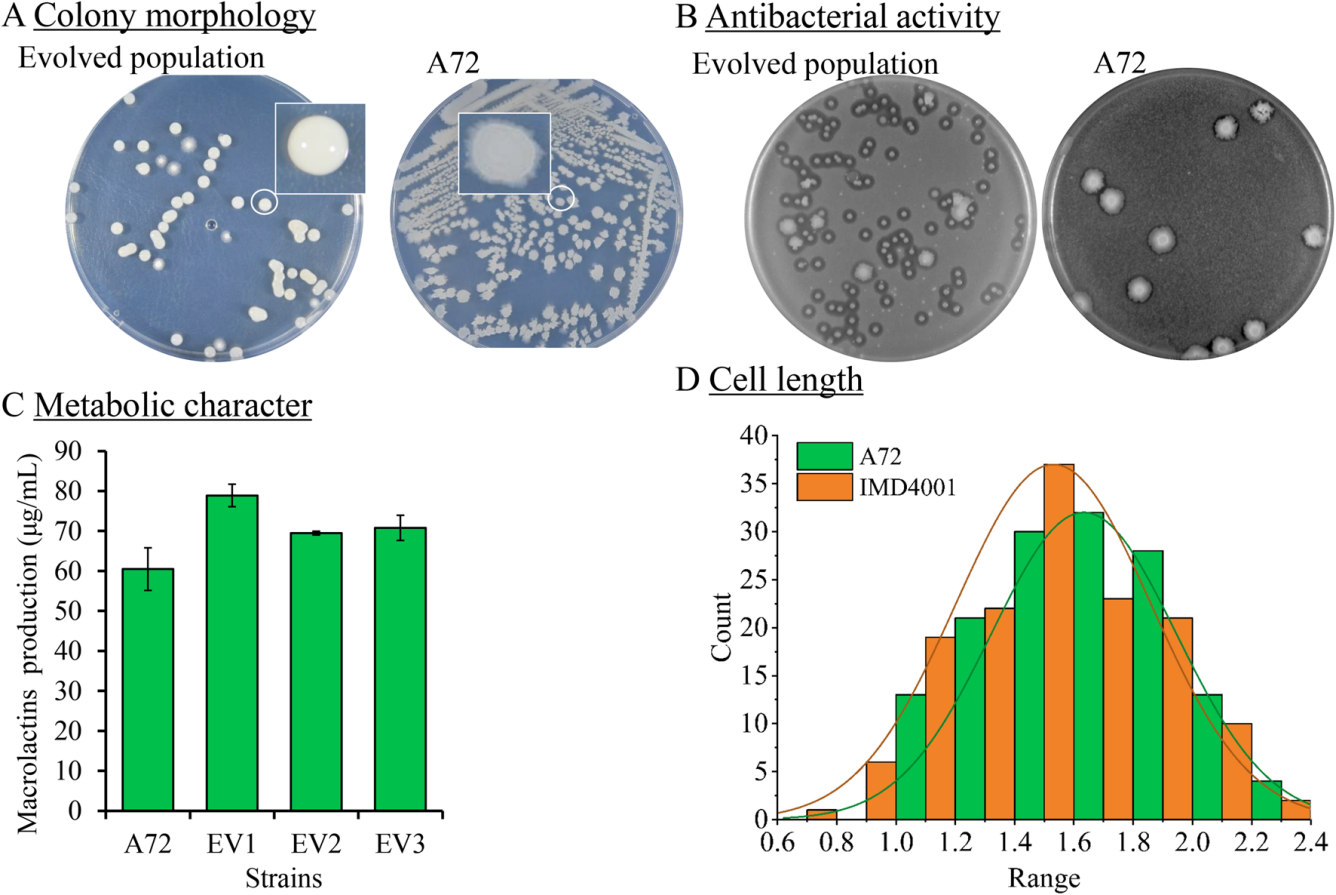
Morphological and physiological characteristics of evolved strains. **A** Comparison of colony morphology between evolved population and parental strain. Overnight culture was spread on plates cultured at 30 °C, photographs were taken after 24 h. **B** Comparison of antibacterial activity. Diluted strains were spread on plates mixed with *Sporisorium scitamineum*, cultured at 30 °C for 24 h, and photographed. **C** Randomly selected mutants exhibited enhanced macrolactins production over the parental strain. Strains were cultured in ISP–50 medium at 30 °C for 24 h, products were measured by HPLC, values and error bars represent mean and standard deviation values (n = 3 cultivations). **D** Cell sizes are reduced in the evolved strain under non-stress conditions. Photomicrographs were performed with a scanning electron microscope. Image-Pro (Plus 6.0) was used to measure the cell size by counting 140 cells in each strain

At the 132^nd^ transfer (approximately 60 days of successive evolution), there was minimal cell growth when the saline concentration was increased to 9% (w/v). At this point, we terminated the ALE experiment. The final mutant population corresponding to the 132nd passage was spread onto ISP-2 agar plates, and from the plating, we found two types of colony morphologies on the plates (Fig. 2A). Of the colonies, 7 of 51 (13.7%) showed a flat, rough, and serrated edge, which is similar to A72, and 44 of 51 (86.4%) were smooth, moist and viscous, milky white. Using ALE to engineer an interesting phenotype, bigger-sized colonies on the screening plates were usually selected [31]. The final concentration of stress factor used for the ALE experiment has usually been used for selecting desired mutants (e.g., caffeine- or ethanol-resistant strains) from the evolved population [25, 32], which leads to our belief that it is easier to select the best-performing mutants at a higher saline level. Thus, to obtain desire mutant strains, the 132nd passage mutant population was spread onto ISP-50 medium plates supplemented with 8% (w/v) saline stress. However, there was not a significant difference in the colony size among the mutants, and as such, it was difficult to select the most robust strains based on colony size under saline stress.

In our previous research, we discovered MLNs showed inhibitory activity against the germination of *Sporisorium scitamineum* [33]. Thus, a bacteriostatic circle test was performed to select the most robust mutant based on the bacteriostatic circle size (Fig. 2B). According to the bacteriostatic circle test results, the mutant population showed increased activity against *S. scitamineum* as compared with the parental strain. However, it was still difficult to select the best mutant strain because all bacteriostatic circle sizes were about the same size. The bacteriostatic circle test prompted us to speculate that the mutant population might be derived from a common saline-resistant cell. Thus, three colonies, labeled EV1, EV2, and EV3, were randomly selected from the mutant population for metabolic analysis by HPLC. After 24 h of fermentation in ISP-50 medium at 30 °C with a rotation of 200 rpm, the EV1 strain showed the highest production of MLNs among the three strains selected (78.89 ± 2.81) μg/mL, which was a 30.46% improvement compared with A72 (Fig. 2C). Since we lacked another feasible strategy to select the best strain from mutant population, we subsequently selected EV1 as the best mutant strain and was renamed IMD4001. According to the photomicrographs, the cell length was reduced in IMD4001 as compared with the parental strain (Fig. 2D, Additional file 1: Fig. S2). The 16S rRNA gene sequencing results showed that IMD4001 has 100% similarity with A72, indicating that IMD4001 was derived from the parental strain A72 (Additional file 2). Genetic stability is generally an important consideration for microbial breeding by mutagenesis methods. In this study, the genetic stability of IMD4001 was validated by 20 generations of continuous sub-cultivation in ISP2 medium.

Based on the results, it was reasonable to deduce that all of the strains with a colony morphology change, which accounted for 86.4% of evolved population, had a positive mutation. However, further studies are needed to validate that these changes are regulated by a genome mutation. In summary, although we lacked an effective selecting strategy, we successfully engineered a mutant strain that had enhanced the production of MLNs via ALE using saline as the screening pressure.

### Characteristics of evolved mutant strain IMD4001

**Fig. 3:**
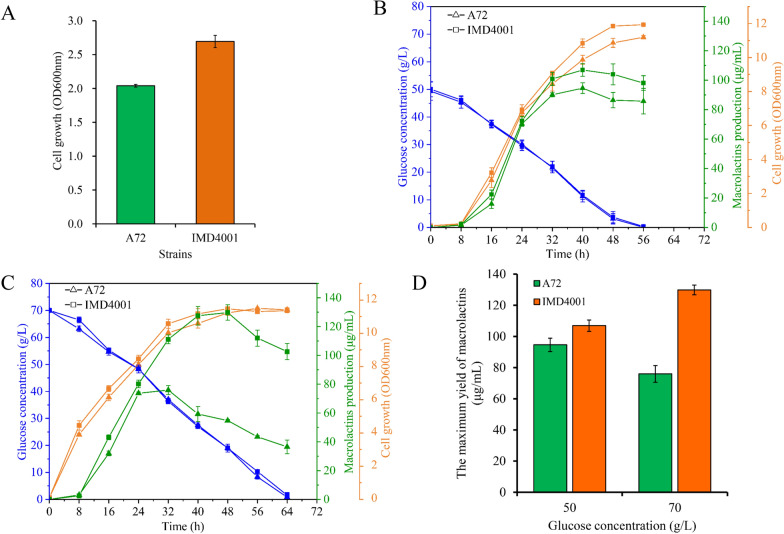
Characteristic analysis results of the parental strain and the mutant strain IMD4001. **A** Saline stress tolerance analysis. The strains were cultured in LB medium in the presence of 6.0% saline. The biomass yield (OD600nm) of the strains was measured at 24 h. Strains were cultivated in ISP-50 **B** or ISP-70 **C** medium in fed-batch fermentation over 56 h, macrolactin production is indicated in green, cell growth is shown in orange, and glucose concentration is shown in blue. **D** The maximum yield of macrolactins of the two strains under different cultural conditions. Values and error bars represent the mean and standard deviation value (n = 3 cultivations)

Saline stress tolerance testing was performed by detecting growth kinetics in the presence of 6.0% (w/v) saline. IMD4001 showed a 32.03% improvement in saline tolerance as compared to the parental strain (Fig. 3A). To test the fermentation characteristics of the evolved strain, IMD4001 and the parental strain A72 were subjected to fermentation tests in flasks. Indices, including growth curves, glucose consumption, and MLNs production from IMD4001 and A72, were compared. It should be noted that the inoculum used was ISP-50 medium containing 3% (w/v) sea salt and 50 g/L glucose, with slight osmotic stress or no stress. As shown in Fig. 3B, D, the MLNs production of IMD4001 (106.89 μg/mL) showed a 13.0% improvement as compared with A72 (94.60 μg/mL), and the biomass of IMD4001 was slightly higher than that of A72. We speculated that the improvement in saline stress tolerance would confer on IMD4001 better performance in other osmotic stresses, including glucose. Thus, fermentation capacity of parental strain and IMD4001 was evaluated under 70 g/L glucose. As shown in Fig. 3C, IMD4001 produced 129.82 μg/mL MLNs, which was approximately 1.71-fold higher than A72. To our surprise, when the glucose concentration in the inoculum was increased from 50 to 70 g/L, IMD4001 showed a 21.5% MLNs production improvement; however, A72 showed a decrease (from 96.6 to 75.9 μg/mL). These results confirmed our hypothesis that strains with saline resistance exhibit enhanced MLNs production, and it was feasible to engineer strains with enhanced MLNs production by ALE with saline stress as the screening pressure. Additionally, as shown in Fig. 3C, the MLNs concentration of A72 started dropping after 24 h of cultivation. MLNs skeleton synthesis is a process of successive condensation of activated malonyl-CoA [34, 35], which is similar to the fatty acid synthesis, and thus we deduce that MLNs also have a catabolism process that is similar to fatty acid oxidation, which could provide additional ATP for cells to cope with stress [36]. In our opinion, 70 g/L glucose is an osmotic stress for A72. In this condition, a large amount of ATP is needed to maintain the cytoplasmic osmotic balance, driving A72 to rely more on MLNs catabolism (or oxidation) for ATP production.

**Fig. 4:**
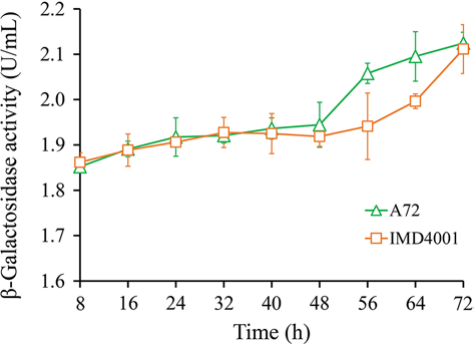
ONPG test results. β-Galactosidase activity was monitored by an increased absorbance at 420 nm. Membrane permeability of A72 is shown as green hollow triangles and that of IMD4001 is shown as orange hollow diamonds. Values and error bars represent the mean and standard deviation value (n = 3 cultivations)

β-Galactosidase catalyzes the O-nitrophenyl-beta-D-galactopyranoside (ONPG) hydrolyze to O-nitrophenol (ONP), which is yellow and absorbs maximally at 420 nm. To investigate the cytoplasmic membrane permeabilization of cells, β-galactosidase release was measured from the supernatant using the ONPG test [37, 38]. The absorption value at 420 nm was used as an indication of membrane permeabilization. The presence of ONPG analysis was performed to test the membrane permeabilization of the evolved strain and parental strain during fermentation using 50 g/L glucose as a carbon source (Fig. 4). The yield of MLNs increased continuously during 8–40 h of culture (Fig. 3B, C), while the degree of cell membrane permeabilization was similar for both strains. The maximum yield of MLNs was achieved at 40 h, and starting at this time point, the degree of cell membrane permeabilization increased in both strains, but A72 was obviously worse than IMD4001. The results implied that IMD4001 had obtained some mechanism to protect cells from serious damage during the late fermentation stage.

### RNA sequencing analysis of global gene expression patterns in ALE strain

**Fig. 5:**
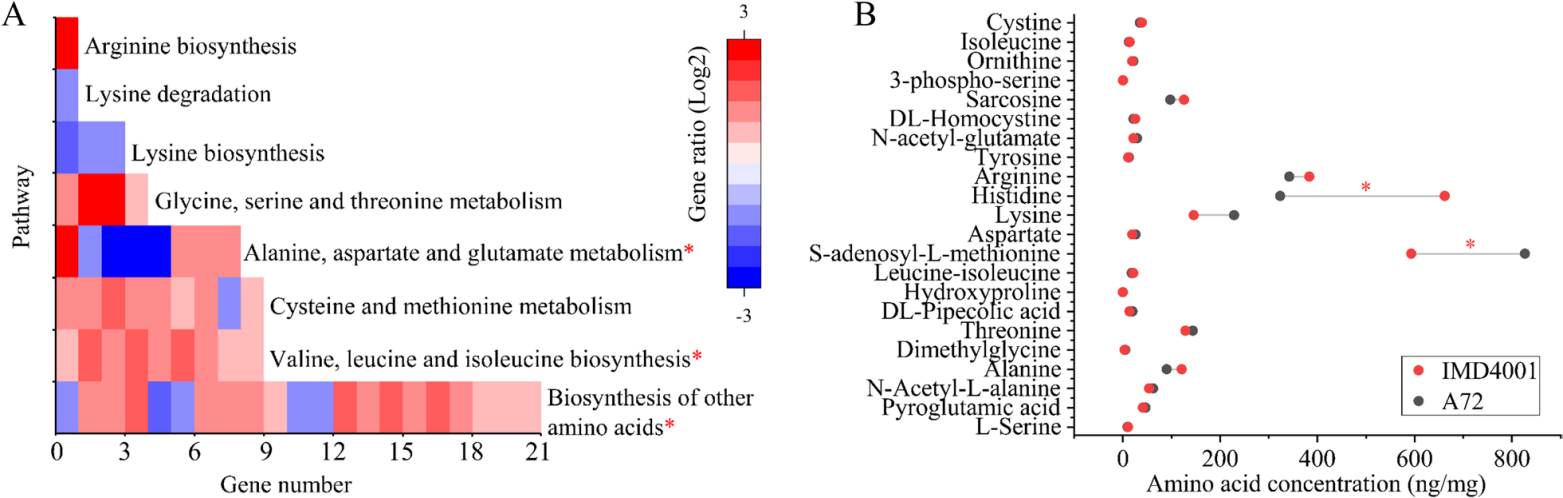
Amino acids metabolism analysis of evolved strain IMD4001. **A** Amino acid pathway enrichment. KEGG pathways enriched in the genes upregulated or downregulated (log2 fold change > 2.0) in evolved strain IMD4001, as compared with A72. **B** Changes in the intracellular amino acid metabolism in IMD4001. Cells cultured in ISP-50 medium for 24 h were harvested for RNA sequencing or metabolomics analysis. Each sample had three replicates; * q < 0.05

**Fig. 6:**
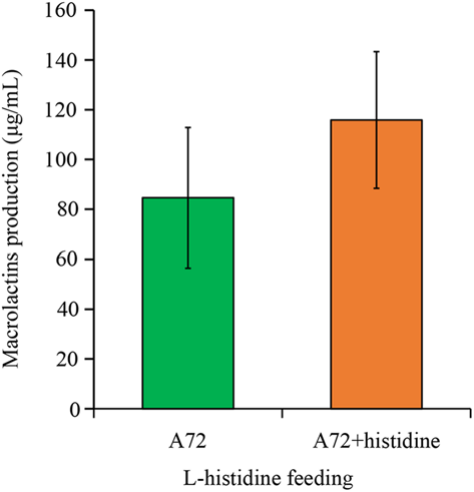
Influence of L-histidine on macrolactins production. Strains were cultivated in ISP-50 medium supplemented with 0.4% L-histidine for 40 h. Values and error bars represent the mean and standard deviation (n = 3 cultivations)

To reveal gene expression changes, which would play a critical role in the development of the desired mutant strain, both IMD4001 and the parental strain A72 were subjected to transcriptome sequencing. Based on the MLNs yield (Fig. 3B), it was observed that the yield of MLNs between IMD4001 and A72 was different at 24 h. Thus, samples were collected for RNA-seq analysis at 24 h. The results showed that IMD4001 had 342 genes that were significantly differentially expressed (log2 fold change > 2, q < 0.05) (Additional file 3). Among these 343 significant DEGs, 155 DEGs were upregulated and 188 DEGs were downregulated. To gain insight into the molecular mechanism underlying the improvement of MLNs production in the evolved strain, GO analysis was used. GO analysis results showed that a certain percentage of the DEGs were involved in a membrane component, including “membrane,” “membrane part,” and “membrane-enclosed lumen” (Additional file 3). Aside from the GO analysis, we also focused on the metabolic pathways that the DEGs were involved in; hence, all of the DEGs were submitted to the KEGG database. A total of 97 pathways from the KEGG database were enriched. Fourteen pathways involving amino acid, nucleotide, carbohydrate, cofactors, vitamins, terpenoids, and polyketides metabolism were significantly enriched (q < 0.05) (Additional file 3). In particular, at least 7 pathways involving amino acid metabolism were enriched (Fig. 5A). We initially speculated that amino acid metabolism plays a critical role in improving the production of MLNs in IMD4001. To further validate the molecular mechanism underlying the strain improvement, the levels of 34 amino acids in IMD4001 and A72 were tested using LC–MS/MS (Additional file 4). Compared with A72, 9 amino acids concentration were increased in IMD4001 (amino acids concentration increase ratio > 1.2), among which the concentration of histidine increased 2.04-fold, whereas 11 amino acids concentration were decreased in IMD4001 (amino acids concentration decrease ratio < 0.8) (Fig. 5B). To validate the influence of amino acids on MLNs production, an L-histidine feeding experiment was performed (Fig. 6). In the presence of 0.4% L-histidine, the production of MLNs in A72 improved by 36.90% as compared with A72 fermentation in ISP-50 without L-histidine for 40 h. These results further implied that amino acids, particularly L-histidine metabolism, may play a critical role in improving the production of MLNs and saline resistance.

### Genome sequencing for identification of mutations causal for improved MLNs production

**Fig. 7:**
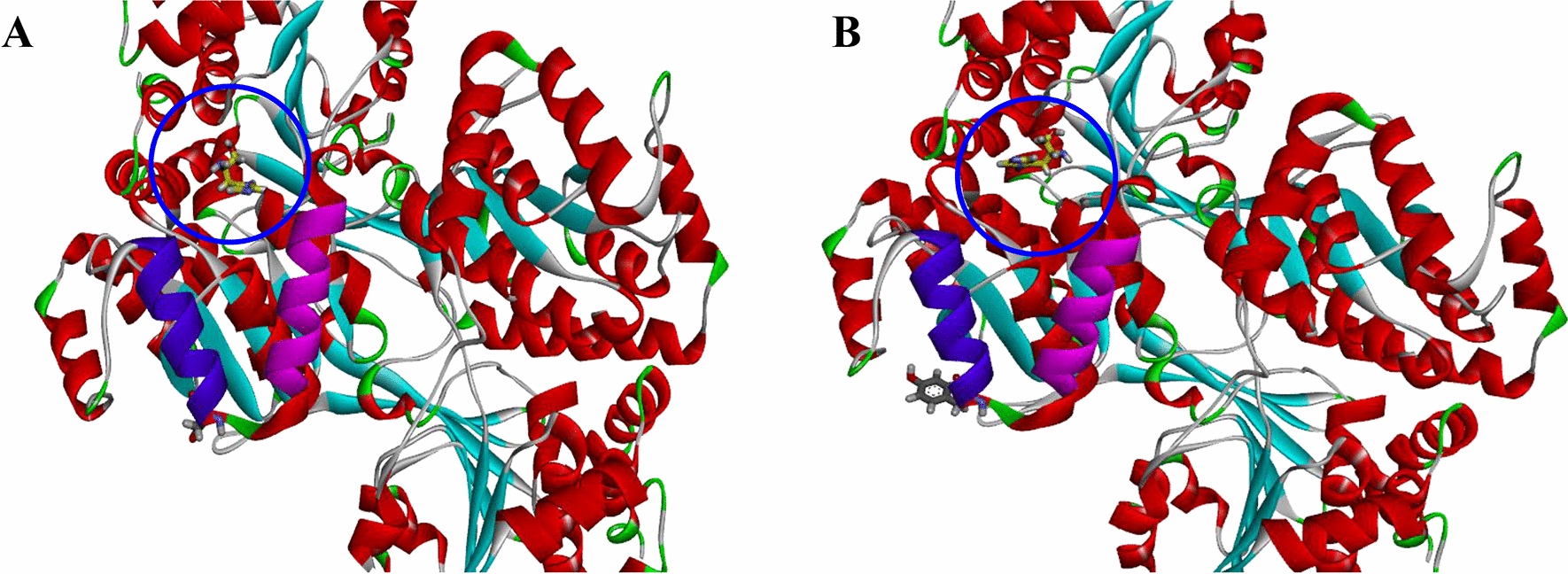
Structural analysis of the SNP mutation HisD^D41Y^. Change in the conformational loop of *hisD* in the wild-type (A) and mutant (B) strains. The structure was modeled by the Discovery Studio 4.1 program. The mutation site is marked by a blue circle

**Fig. 8:**
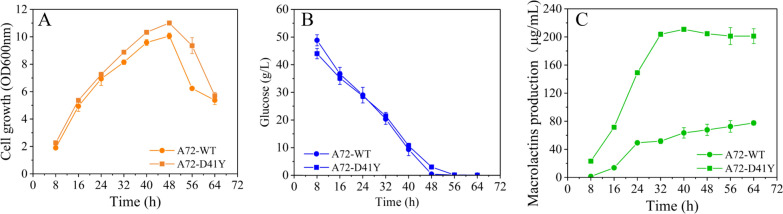
Influence of a D42Y mutation in *hisD* on macrolactins production. To validate the influence of a D42Y mutation in *hisD* on macrolactins production, *hisD*^D41Y^ and *hisD* were overexpressed in A72, yielding A72-WT and A72-D41Y, respectively. The cell growth (**A**), glucose consumption (**B**), and macrolactins production **C** of the two strains were compared. The plasmid pHT3101 was used as a skeleton for gene overexpression. Strains were cultivated in ISP-50 medium supplemented with 0.5 μg/mL erythromycin. Values and error bars represent the mean and standard deviation, respectively (n = 3 cultivations)

**Fig. 9:**
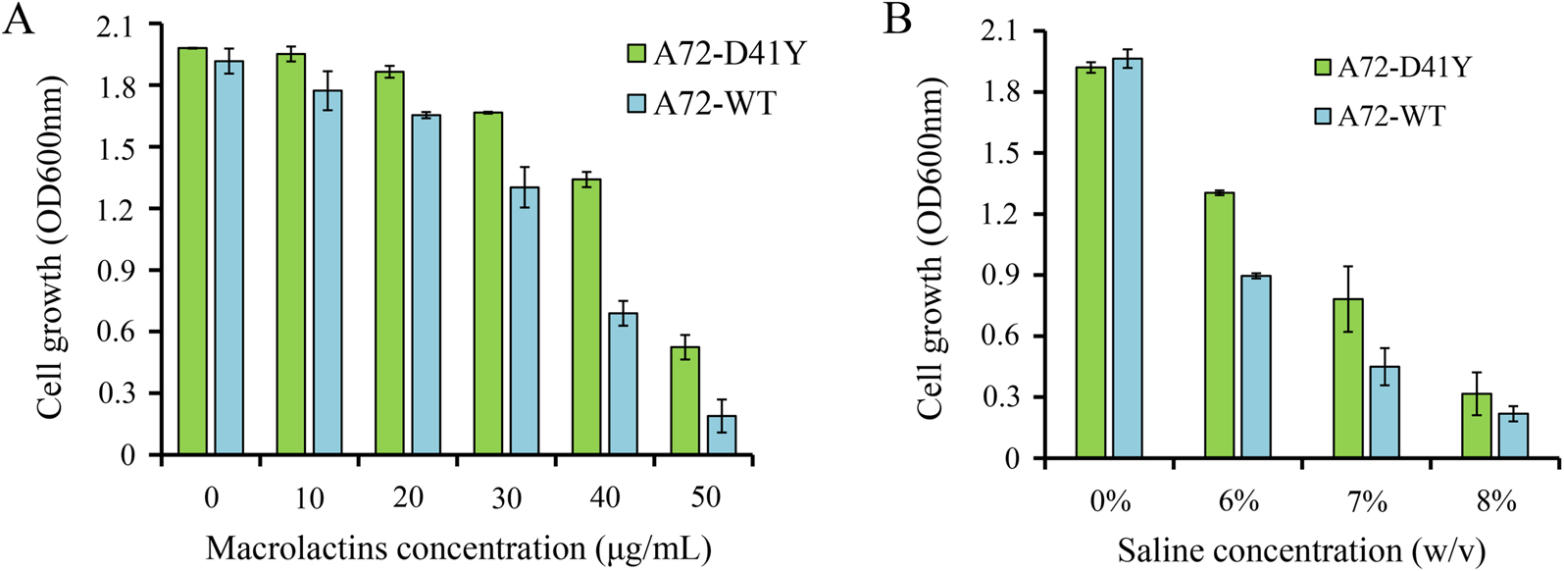
Stress tolerance comparison of *hisD*/*hisD*^D41Y^–overexpressing strains. **A** Comparison of macrolactins resistance between *hisD*–overexpressing strain (A72–WT) and *hisD*^D41Y^–overexpressing strain (A72–D41Y). Seed cells were inoculated in LB medium supplemented with 10–50 μg/mL macrolactins (without erythromycin), cultured at 30 °C in a shaker at 200 rpm. After incubating for 12 h, the OD600nm values of the cultures were measured. **B** Comparison of saline stress tolerance between A72–WT and A72–D41Y. Seed cells were inoculated in LB medium supplemented with 6–8% (w/v) saline (without erythromycin), and then cultured at 30 °C in a shaker at 200 rpm. After incubating for 12 h, the OD600nm values of the cultures were measured. Values and error bars represent the mean and standard deviation, respectively (n = 2 cultivations)

Apart from the gene expression profile of the evolved strain, we were also interested in looking at the genomic variations. To identify any genomic variations, whole-genome sequencing was performed on IMD4001. The whole genome sequencing of IMD4001 produced 1.14 Gb of Pacbio long reads (287 ×) and 1.13 Gb (285 ×) of Illumina short reads. The short reads was produced on the Illumina Novaseq 6000 sequencer using the pair-end technology (PE 150). Following alignment to the genomic sequence of A72, a total of five mutations were identified in IMD4001 (Additional file 3). Among the identified mutations, two “insertion” mutations were found within the intragenic space, two InDels within open reading frames (ORFs) were found, which caused frameshift mutations in two genes, and a single nucleotide polymorphism (SNP) causing a nonsynonymous amino acid change was located in the *hisD* gene, which encodes a histidinal dehydrogenase. Combined with the RNA-seq results, we deduced that the mutation in *hisD* might be the mutation responsible for improving the MLNs production in the mutant, and it was selected for further investigation. Mapping with the reference genome, the 121^st^ nucleotide of *hisD* was mutated from G to T, and the corresponding amino acid was transformed from Asp (GAC) to Tyr (TAC). Analysis of the 3D structure of HisD ^D41Y^ by Discover Studio 4.1, revealed that the mutation site was located at the loop of Lys42-Asp52 and Lys27-Arg37 (Fig. 7). To validate if the identified mutation *hisD* ^D41Y^ was causal for improved MLNs production, the wild-type and *hisD* mutant (*hisD*^D41Y^) were overexpressed in the parental strain A72, and the fermentation tests in ISP-50 medium were performed (Fig. 8). Compared with the control strain A72-WT, A72-Y41D showed a 3.42-fold increase in MLNs production, in addition to a slight cell growth improvement. To explain the relationship among saline stress tolerance, end-product resistance, and MLNs production improvement in this study, the MLNs resistance and saline stress tolerance of A72-WT and A72-D41Y were investigated (Fig. 9). We compared growth profiles between A72-WT and A72-D41Y in culture medium with different MLNs levels (from 10 to 50 μg/mL; Fig. 9A). There was a significant difference in cell growth between the strains under MLNs conditions, and the difference increased with increasing MLNs level. A72-D41Y also showed an improvement in saline tolerance compared with A72-D41Y (Fig. 9B). Results showed that strains with MLNs production improvement exhibited an increase in saline stress tolerance and MLNs resistance simultaneously. Combining the metabolic and genome sequencing results, we deduced that *hisD* ^D41Y^ was likely a key contributor to the improvement in MLNs production in IMD4001.

## Discussion

The objective of this study was to create a mutant strain with enhanced MLNs production. Used saline with a gradual increase in concentration as screening pressure, we obtained a saline-resistant mutant that exhibited enhanced production of MLNs via ALE successfully. To gain insight into the molecular mechanisms of saline resistance and MLNs production improvement in *B. siamensis*, comparative analyses of the mutant IMD4001 strain and parental A72 strain were performed at the physiological, transcriptomic, and genetic levels.

### Auxiliary device was necessary for ALE

Although ALE is a powerful tool for creating a target microbial phenotype, it is usually a time-consuming process. For instance, Mo et al. spent 100 days developing an ethanol-tolerant strain via manual ALE, and the process involved subculturing 450 times in flasks [25]. Wang et al. also spent 84 days enhancing the ferulic acid tolerance of *Yarrowia lipolytica* [20]. Hence, ALE is tedious and laborious when using this process to engineer a microorganism with a specific phenotype. Additionally, there is always an inherent risk of contamination when subculturing frequently in flasks, even in a sterile environment. Furthermore, since cell growth is usually used as an indicator of whether a strain with the target phenotype has developed in the parental strain, contaminating bacteria that are resistant to the screening pressure can influence the judgment of the researchers and potentially cause the entire ALE experiment to fail. Thus, avoiding microbial contamination is a top priority during ALE. Based on these considerations, using an assisting device is important for ALE experiment. In this study, we assembled a simple device assisting strain evolution by applying ALE (Fig. 1). With the help of the device, the whole evolution process was almost operated in a closed environment except the “a” container, which was refilled with fresh medium. The closed environment effectively avoided contamination. Moreover, although it still took an extended period (60 days) to create IMD4001 with multiple subculturing (132 times), the subculture operated via the self-made device, which greatly reduced the labor. Thus, the ALE device assembled in this study was labor-saving and contamination-reducing and was also successful in creating a microorganism with the targeted phenotypes.

### Using saline as screening pressure to create a strain with enhanced production of MLNs

To the best of our knowledge, this is the first report on creating an excellent antibiotic production strain by using ALE with saline as the screening pressure. Yi et al. applied ARTP mutant technique to develop a robust MLNs-producing strain[30]. According to the change in colony morphology after ARTP treatment, an excellent mutant strain was obtained with a low-screening efficiency of 6.67%. However, this screening strategy could not be applied to the next round of mutant treatment, since the colony morphology did not change after the first round of mutation. To overcome these limitations, we used saline as the screening pressure to create a strain with increased MLNs production via ALE. During the process, evolution was continuously performed until the strains did not grow in a specific saline concentration, and an 86.4% positive mutant proportion was obtained after 60 days of successive batch culture. However, it was still a limitation in this study, and the lack of an effective strategy to select the best performing mutant strains from the evolved population was also a limitation.

During the fermentation process, strains often suffer inhibition from environmental stress. To release such inhibition, a specific environmental stress is selected as the screening pressure via ALE, including lignocellulose-derived inhibitor resistance [39–41], thermal stress resistance [42, 43], and end-product resistance [44, 45]. In this study, we choose saline stress, which is not part of the fermentation process, as the selective pressure to create a strain with enhanced production of MLNs. The correlation of two independent phenotypes to improve MLNs production by ALE was innovative and provided insights for improving the biosynthesis production of other antibiotics or toxic compounds.

### Amino acids were critical to the improvement in MLNs production

A previous study revealed that antibiotic biosynthesis was enhanced when histidine and valine were combined with glutamic acid as nitrogen sources in the fermentation medium[46]. Consistent with our study, an increased extracellular histidine concentration significantly improved MLNs production (Fig. 6). In *Saccharopolyspora erythraea*, precursors for macrolides synthesis were supplied from branched-chain amino acid degradation pathways and other catabolic pathways [47, 48], and macrolides production was strongly influenced when the flux of precursor metabolites was increased [49, 50]. Additionally, Liu et al. identified that the regulator, SACE_Lrp, which regulates the transportation and catabolism of branched-chain amino acids, was enhanced in the presence of histidine [51]. Thus, amino acids provide precursors for macrolides biosynthesis, which explains the change in the amino acid metabolism in this study (Fig. 5).

Antibiotic accumulation usually results in feedback inhibition on the producer strain via inhibiting enzyme activity and DNA intercalation [13]. As shown in the ONPG test (Fig. 4), the cell membrane of A72 endured more damage than IMD4001 beginning at 40 h, which demonstrated that IMD4001 developed its own mechanism to protect against environmental stress. Thus, we inferred that amino acids are not only used as a nutrient or precursor for macrolides synthesis but also play an important role in self-resistance in this study. Both glycine-betaine and proline have been reported to function as osmo-protectants in several bacteria [52]. Aside from its use as a protectant, the histidine decarboxylation pathway generates metabolic energy and feeds the proton-motive force for the survival of strains under energy-limiting conditions [53]. Osmo-protectants have been shown to reduce the susceptibility of strains to antibiotics [54, 55]. Thus, it was reasonable to deduce that amino acid metabolism in this study was able to release the feedback inhibition in IMD4001, allowing for increased production of MLNs. Microorganisms have also evolved other sophisticated transport mechanisms to release feedback inhibition; for example, ATP-binding cassette (ABC) transporters have been shown to facilitate the efflux of toxic compounds from the cells, which have been conferred resistance to macrolides [56–58]. RNA sequencing analysis in this study found 11 DEGs involved in ABC transporters, participate in antibiotic, lantibiotic, glycine-betaine, choline, carnitine, and phosphate transportation (Additional file 3). Therefore, the improvement in MLNs production in the evolved strain IMD4001 may also be attributed to the enhancement in its transmembrane transport ability (Additional file 4).

Based on the comparative genome analysis results, we successfully identified a non-synonymous mutation in the *hisD* gene (*hisD*^D41Y^), which was identified to be linked to MLNs production, saline stress tolerance, and MLNs resistance improvement. By overexpressing *hisD*^D41Y^, A72-Y41D showed a 3.42-fold increase in MLNs production (Fig. 8), 1.94-fold increase in MLNs resistance under 40 μg/mL MLNs (Fig. 9A), and 1.74-fold increase in saline tolerance under 7% saline level (Fig. 9B) compared with A72-WT. These results obviously revealed a positive correlation among the three phenotypes. Thus, in this study, it was reasonable for us to deduce that the increasing saline stress tolerance obtained from ALE using saline as screening stress, conferred IMD4001 resistance to MLNs, and eventually led to an improvement in MLNs production, although further studies are needed to validate the speculation. To our knowledge, the function of SNP *hisD*^D41Y^ in MLNs production, resistance, and saline stress tolerance improvement has not been reported previously. Thus, it is insightful to deduce the rational metabolic engineering to further improve macrolides production.

## Conclusions

In the present work, ALE technology was applied to enhance MLNs production of *B. siamensis* A72, using saline stress as selecting pressure. As a result, a robust strain IMD4001, which showed saline stress tolerance, MLNs resistance, and MLNs production improvement simultaneously, was obtained after 60 days successive evolution. The fermentation character shown that IMD4001 produced MLNs with 11.93% and 71.04% improvement in non-saline stress medium ISP-50 and ISP-70, respectively, as compared with the parental strain. The results of transcriptome and metabolome indicated amino acid metabolism played a critical role in MLNs production improvement. Based on the whole genome sequencing results and overexpressing experiment, mutation in *hisD* (D41Y) was found to benefit MLNs production improvement. Our strategy using in this study might be applicable to improve the production of other kinds of macrolide antibiotics and other toxic compounds. Moreover, the identification of the *hisD* mutation will allow for the deduction of metabolic engineering strategies in future research.

## Materials and methods

All plasmids, oligonucleotide primers and strains used in this study were listed in Additional file 2.

### Strain, medium and cultivation conditions

A72 is a MLNs-producer strain [30], which was used as the parental strain for ALE. International *Streptomyces* Project (ISP) medium 2 (ISP-2) (number represents glucose concentration) containing 2 g/L yeast extract, 2 g/L malt extract, 3% (w/v) sea salt, and 2 g/L glucose at pH 6.5, was used for the cultivation of seed cells. ISP-50 or ISP-70, containing 10 g/L yeast extract, 6 g/L malt extract, 3% (w/v) sea salt, and 50 g/L or 70 g/L glucose at pH 6.5, was used for fermentation character tests. ISP medium-50-S (letter “S” represents medium supplement with 5%–10% (w/v) saline) containing 10 g/L yeast extract, 6 g/L malt extract, 5–10% (w/v) sea salt, and 50 g/L glucose at pH 6.5 was used for ALE experiments. ISP-50 supplemented with 0.5 g/mL erythromycin was used for gene overexpression tests. *Escherichia coli* strain Trans1-T1 (TransGen Biotechnology Co., Ltd, Beijing, China) used for plasmid construction was cultured at 37 °C in Luria Broth (LB) medium supplemented with the appropriate antibiotics. A72 cultivation was carried out at 30 °C with a rotation of 200 rpm.

### ALE for improving saline tolerance

Previous study revealed that combined multiple mutagenesis strategy (e.g., UV mutagenesis, ARTP, or ALE) had higher efficiency than that of a single one [59, 60]. In addition, the combination of ARTP mutagenesis and ALE has been successfully applied to breed desired phenotype of strains [61, 62]. In this study, cells undergo two types of DNA mutation, namely, ARTP mutation and adaptive mutation, 6% saline was used to screen the saline stress-resistant mutants from the ARTP-mutagenized population. At this point, the ARTP mutants with saline stress tolerance were enriched, and they were used as the parental strains for the following adaptive experiment. Since there was a low-frequency spontaneous mutation in cell life process, repeating the screening experiment at increasingly higher saline concentrations helped enrich the spontaneous mutation, which is beneficial to strain tolerance improvement.

The ARTP-mutagenized population was used as the initial cell pool for evolving saline-resistant mutants by ALE, using saline as the screening pressure. First, the initial saline concentration (x%) used in the evolutionary selection experiments was determined. A72 was inoculated into 5 mL of ISP-50 containing a gradient concentration of saline in a 50-mL, screw-cap tube to an initial optical density at 600 nm (OD600nm) of 0.1 and cultured at 30ºC at 200 rpm. After incubating for 24 h, the OD600nm values of the cultures were measured by a visible spectrophotometer (Jingke 721G-100, Shanghai, China). A72 growth was inhibited at 6% (w/v) stress level (Fig. S1), which was then selected as the initial concentration for evolutionary screening. To reduce the labor intensity of ALE, a simple device was assembled (Fig. 1). During the ALE experiment process, batch cultivation mode was selected, which was operated as follows: The ARTP-mutagenized cells were inoculated into the “b” container with 30 mL of fresh ISP-50-S to an initial OD600nm value of approximately 0.3, cultured at 30ºC, and dissolved in oxygen by magnetic bar stirring. When cells passed at exponential phase of growth in the “b” container (the OD600nm value reached 5.0–8.0), approximately 25 mL of the culture was pumped out of the “b” container, and 1 mL of the culture was stored in 30% (v/v) glycerol at − 80 °C. To obtain the next passage, a total of 25 mL of fresh ISP-50-S medium from the “a” container was supplemented through Pump 2 at one time, and cultured at 30ºC until the OD600 value reached 5.0–8.0. After approximately 40 generations of batch cultivation, the ISP-50-S medium (1000 mL) in the “a” container was exhausted, and the container was then refilled with sterile ISP-50-S with the saline concentration increased by 1%, which was then used for the next stress level for screening. When the cells could no longer grow at a specific saline concentration in ISP-50-S medium, the ALE experiment was terminated.

The whole ALE experiment was monitored. At the 30th transfer, biofilm formation was observed on the vessel “b” wall. The OD600nm values of the cultures were measured by a visible spectrophotometer (Jingke 721G-100, Shanghai, China). A microscope (Shunyu L3230, Shanghai, China) was used to monitor cell morphology, contamination, and cell crash during the evolving process, especially when increasing the saline concentration.

### 16S rRNA gene sequencing

To explain the genetic relationship between A72 and IMD4001, the 16S rRNA gene was sequenced. Primers 27 F and 1492 R were used to amplify the 16S rRNA gene via PCR using chromosomal DNA of A72 and IMD4001, respectively. The fragments were gel purified, and given to Genedenovo Biotechnology Co., Ltd. (Guangzhou, China) for sequencing. The sequencing results were submitted to the EzBioCloud Database (https://www.ezbiocloud.net/tools/pairAlign) for gene blast.

### Plasmid construction for *hisD* overexpression

For overexpression of the *hisD* gene, pHT3101 was used as the backbone plasmid, which was kindly provided by Prof. Wenli Li (Ocean University of China, Qingdao, China). The backbone linear fragment was amplified by using primers pHT3101 F and pHT3101 R. Primers hisD F and hisD R were used to amplify the wild-type and mutant *hisD* gene cassette via PCR using chromosomal DNA of A72 and IMD4001 as template, respectively. The fragments were gel purified, assembled, and transformed into *E. coli* competent cells according to the manufacturer’s instructions of pEASY-Basic Seamless Cloning and Assembly Kit (TransGen Biotech Co., Ltd., Beijing, China). Transformants were selected on LB plates (pH value 7.0) supplemented with 100 μg/mL erythromycin. Plasmids were transformed into A72 using the following electroporation parameters: voltage, 900 V; capacitance, 50 μF; resistance, 200 Ω; and cuvette, 2 mm. Transformants were selected on LB plates supplemented with 0.5 μg/mL erythromycin. The plasmids pHT3101-WT and pHT3101-D41Y were transformed into A72 to yield A72-WT and A72-D41Y, respectively.

### Determination of cell morphology

Single colonies of A72 and IMD4001 from agar plates were inoculated in the seed medium and cultured at 30 °C in a shaker at 200 rpm. After 15 h, the cells were harvested by centrifugation, transferred into 20 mL of fresh seed medium in 50-mL flasks to an OD600nm of 0.1, and incubated at 30 °C in a shaker at 200 rpm for 15 h. Cells were then harvested, inoculated into 200 mL of ISP-50 medium to an OD600nm of 0.1, and were incubated at 30 °C in a shaker at 200 rpm for 32 h. Cells were harvested and washed three times with 1 × phosphate-buffered solution (PBS, pH 7.4). Then the cells were dehydrated by an ethanol gradient (e.g., 30%, 50%, 70%, 80%, 90%, and 100% (v/v)), each for 7 min. The cells were incubated in butyl alcohol at room temperature for 5 min, and the pellets were then freeze-dried at − 80 °C. The pellets were then given to Shuosibai Testing Technology Co., Ltd. (Wuhan, China) for further preparation and analysis. Photomicrographs were imaged with a scanning electron microscope (SEM) (Zeiss GeminiSEM 300, Oberkochen, Germany). The software Image-Pro (Plus 6.0) was used to measure the cell length, which was completed by counting 140 cells obtained at the same magnification for each strain.

### Stress tolerance analysis of evolved strain

To test the saline stress tolerance, seed cells were inoculated into 20 mL of LB medium in the presence of 6.0% saline in 100-mL flasks and then cultured at 30 °C in a shaker at 200 rpm for 24 h. Cell growth was monitored by measuring the OD600nm.

In our previous study, we found that A72 did not yield MLNs using LB as a growth medium. Thus, to avoid the influence of end-product MLNs and erythromycin (a kind of polyketide, used for maintaining plasmids in *hisD*/*hisD*^D41Y^ overexpressing strains), LB medium without erythromycin was used as the growth medium to study the saline stress tolerance and end-product resistance of *hisD*/*hisD*^D41Y^ overexpressing strains. To characterize the saline stress tolerance, seed cells were inoculated into 2 mL of LB medium supplemented with a gradient concentration of saline [from 6 to 10% (w/v)] in a 24-deep well plate, and then cultured at 30 °C in a shaker at 200 rpm for 12 h. Cell growth was monitored by measuring the OD600nm values. For characterizing the MLNs resistance, seed cells were inoculated into 2 mL of LB medium supplemented with a specific MLNs concentration (10 or 30 μg/mL) in 24-deep well plate, and then cultured at 30 °C on a shaker at 200 rpm for 12 h. Cell growth was monitored by measuring the OD600nm values.

### Fermentation capacity analysis of evolved strain

In our previous study, the concentration of components in ISP-2 medium and other parameters influencing the MLNs production of A72 were optimized. The bacterium grown aerobically in ISP-50 medium (pH 6.5) showed maximum MLNs production, at 30 °C in a shaker at 200 rpm.

To test the fermentation performance of the evolved strain, seed cells were inoculated into 200 mL of fermentation medium in 500-mL flasks. ISP-50 medium (pH value 6.5) was used for testing the fermentation capacity under normal conditions. ISP-70 medium (pH 6.5) was used for testing the fermentation capacity under hyperosmotic conditions, and ISP-50 medium (pH 6.5) supplemented with 0.5 μg/mL erythromycin was used for testing the fermentation capacity of strains by *hisD* overexpression. The fermentation process was performed at 30 °C at 200 rpm. Cell growth was monitored by measuring the OD600nm. The glucose concentration in the fermentation broth was measured by a biosensor analyzer (Institute of Microbiology SBA-40E, Shangdong, China). The method for measuring MLNs production was described previously [30] with some modifications. Briefly, 1 mL of fermentation broth was added to 1.5 mL of methanol. The sample was incubated at room temperature for 10 min and then centrifuged at 10,000 rpm for 20 min. The supernatant was filtered through a 0.22-μm filter. The filtered fermentation broth-methanol mixture (100 μL) was analyzed via HPLC (Waters e2695, Milford, MA, USA) at an ultraviolet wavelength of 227 nm on a C18 column (Waters XB-C18, Milford, MA, USA). Water and methanol were used as the mobile phase with a flow rate of 1 mL/min, and the column was kept at 30 °C.

### ONPG test

To test cytoplasmic membrane permeabilization, IMD4001 and the parental strain A72 were subjected to the ONPG test. The supernatant of 0.8 mL cell cultures was collected by centrifugation at 12,000 rpm for 2 min. Then, 0.5 mL supernatant was mixed with 200 μL of 0.05 mol/L ONPG (Beijing solabao Technology Co., Ltd., Beijing, China) and incubated at 37 °C. After 40 min, 0.25 mL of 0.5 mol/L Na_2_CO_3_ was added to terminate the reaction, and the hydrolysis of ONPG to ONP was monitored at 420 nm by a multimode plate reader (Victor Nivo-PE, PerkinElmer Co., Ltd., Massachusetts, USA).

The β-Galactosidase activity unit calculation is (OD420nm × A)/(B × C × 0.0045), where A is the volume of the reaction mixture (0.7 mL), B the reaction time (40 min), C the sample volume (0.8 mL), and 0.0045 the extinction coefficient.

### The whole genome sequencing

Seed cultures were prepared as mentioned above (“Determination of cell morphology” section). Cells were harvested by centrifugation, inoculated into 200 mL of ISP-50 medium in 500-mL flasks to an initial OD600nm of 0.1, and then cultured at 30 °C in a shaker at 200 rpm. After 32 h, cells from 50 mL of culture broth were harvested in pre-cooled Falcon tubes in liquid nitrogen by centrifuging for 5 min at 4 °C. The pellets were washed twice with normal saline and then frozen in liquid nitrogen for 2 min. Samples were given to Genedenovo Biotechnology Co., Ltd. (Guangzhou, China) for DNA extraction and genome sequencing. The *B. siamensis* KCTC 13,613 genome was used as a reference. Based on the alignment of the genome, it is 96.22% identical between *B. siamensis* KCTC13613 and *B. siamensis*108 (the parental strain of A72). Genomic DNA sequencing of both the parent and evolved strains was performed as described previously [63]. The genome sequencing raw data were deposited in the NCBI Sequence Read Archive under the accession number CP075590.

### RNA sequencing

The samples used for RNA sequencing were prepared as described above (“The whole genome sequencing” section). Total RNA was extracted using TRIzol (Life Technologies, CA, USA). cDNA libraries were sequenced by Genedenovo Biotechnology Co., Ltd. (Guangzhou, China). The *B. siamensis* KCTC 13,613 genome was used as a reference. Bowtie2 (version 2.2.8) was used to map the quality-trimmed reads to the reference [64], identify known genes, and gene expression was calculated by RSEM [65]. An average of 10.15 ± 0.35 million cleaned reads and an average mapping rate of 98.98% ± 0.6% corresponding to the reference genome were generated. The gene expression level was normalized by using the fragments per kilobase of transcript per million (FPKM). Differentially expressed genes (DEGs) were identified with log2 fold change ≥ 2 and q value < 0.05. DEGs were then subjected to Gene Ontology (GO) function analysis and Kyoto Encyclopedia of Genes and Genomes (KEGG) pathways analysis, and q values < 0.05 were used as threshold. The RNA sequencing raw data were deposited in the China National Center for Bioinformation (CNCB) Genome Sequence Archive under the accession number CRA006681.

Calculation of p and q value: Using edgeR software to screen differential expressed genes, the p value of genes was obtained by negative binomial distribution tests, and then the FDR value obtained by BH correction. The p values in GO enrichment analysis and KEGG enrichment analysis were obtained through the hypergeometric distribution test of the foreground value of the KEGG pathway or GO term and background value, and then the Q value obtained through BH correction.

### Quantitative metabolomics

Intracellular amino acids were quantified by using HPLC-AccQ•Tag method [66]. Ethanol extracts were dried at 65 °C until the ethanol evaporated completely, and then the dried extracts were dissolved in 100 μL of water. The amino acid standards and extracts were derived following the AccQ-Fluor reagent kit manufacturer’s instructions (Waters, Milford, MA, USA). The derivatization solutions were filtered through a 0.22-μm filter, and HPLC (Waters e2695, Milford, MA, USA) analysis was performed through AccQ•Tag column (4 μm, 3.9 × 150 mm) with a flow rate of 1 mL/min at 37 °C. The signal was detected at an ultraviolet wavelength of 227 nm. The mobile phase included AccQ•Tag A solvent (A), acetonitrile (B), and water (C). The proportion of the mobile phase was changed as follows: 0–0.5 min, 100% A; 0.5–18 min, 99% A and 1% B; 18–19 min, 95% A and 5% B; 19–29.5 min, 91% A and 9% B; 29.5–33 min 83% A and 17% B; 33–36 min, 60% B and 40% C; and 36–45 min, 100% A.

## Supplementary Information

**Additional file 1. Fig. S1.** Saline stress tolerance of parental strain. Saline ranging from 3 to 10% (w/v) was directly added to the fermentation medium containing 50 g/L glucose, and the growth kinetics was measured. Values and error bars represent the mean and standard deviation (n = 2 cultivations). **Fig. S2.** Scanning electron microscope (SEM) images of parental strain A72 **A** and evolved strain IMD4001 (**B**). Cells were incubated in fermentation medium containing 50 g/L glucose, and cultured at 37 °C for 24 h. Cells were observed at 5 kV and 10,000 × magnification; scale bars correspond to 1 μm.

**Additional file 2: Table S1.** Oligonucleotide primers used in this study. **Table S2.** Plasmids and strains used in this study. 16S rRNA gene sequencing results.

**Additional file 3.** Transcriptome analysis. (Sheet 1) All transcriptome data (3810 different transcripts in total). (Sheet 2) Different expression genes (log2 fold change > 2, q < 0.05). (Sheet 3) Assignment to GO category. (Sheet 4) KEGG Pathway. Genes involving amino acids metabolism from these data were used for the visualization of results presented in Fig. 5A. (Sheet 5) DEGs involving transmembrane transport. (Sheet 6) Accumulated alterations during adaptive laboratory evolution of genome. A file containing DNA mutations in IMD4001 compared to A72.

**Additional file 4.** Intracellular amino acids metabolome analysis. These data were used for the visualization of results presented in Fig. 5B.

## Data Availability

All data generated or analyzed during this study are included in this published article and its Additional files.

## Abbreviations

MLNs: Macrolactins
ALE: Adaptive laboratory evolution
PKS: Polyketide synthase
A72: *Bacillus siamensis* A72
ONPG: O-nitrophenyl-beta-D-galactopyranoside
OD420nm: Optical density at 420 nm
FPKM: Fragments per kilobase of transcript per million
DEGs: Different expression genes
BH: Benjamini and Hochberg
GO: Gene ontology
KEGG: Kyoto encyclopedia of genes and genomes
LC–MS/MS: Liquid chromatograph mass spectrometer
ORFs: Open reading frames
SNPs: Single nucleotide polymorphisms
ARTP: Atmospheric room temperature plasma
ABC: ATP-binding cassette
ISP-2: International streptomyces project medium 2
LB: Luria Broth
OD600nm: Optical density at 600 nm
SEM: Scanning electron microscope
HPLC: High performance liquid chromatography
