# Supplementary material for: Improvement of macrolactins production by the genetic adaptation of Bacillus siamensis A72 to saline stress via adaptive laboratory evolution
Source: Microb Cell Fact. 2022 Jul 19;21:147. doi: 10.1186/s12934-022-01871-9 (PMC9294813; doi:10.1186/s12934-022-01871-9)
Supplement: Supplementary file 1 — Additional file 1. Fig. S1. Saline stress tolerance of parental strain. Saline ranging from 3 to 10% (w/v) was directly added to the fermentation medium containing 50 g/L glucose, and the growth kinetics was measured. Values and error bars represent the mean and standard deviation (n = 2 cultivations). Fig. S2. Scanning electron microscope (SEM) images of parental strain A72 A and evolved strain IMD4001 (B). Cells were incubated in fermentation medium containing 50 g/L glucose, and cultured at 37 °C for 24 h. Cells were observed at 5 kV and 10,000 × magnification; scale bars correspond to 1 μm. [file 12934_2022_1871_MOESM1_ESM.pdf]

Additional file 1

Fig. S1

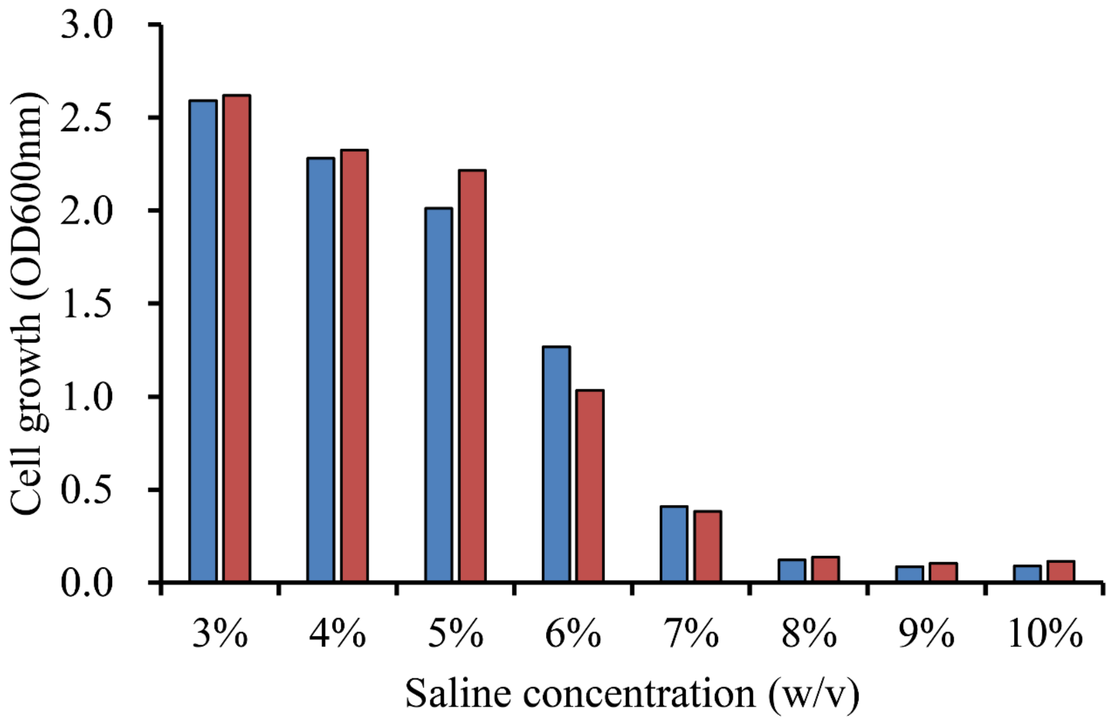

Fig. S1 Saline stress tolerance of parental strain. Saline ranging from 3% to 10% (w/v) was directly added to the fermentation medium containing 50 g/L glucose, and the growth kinetics were measured. Values and error bars represent the mean and standard deviation (n = 2 cultivations).

Fig. S2

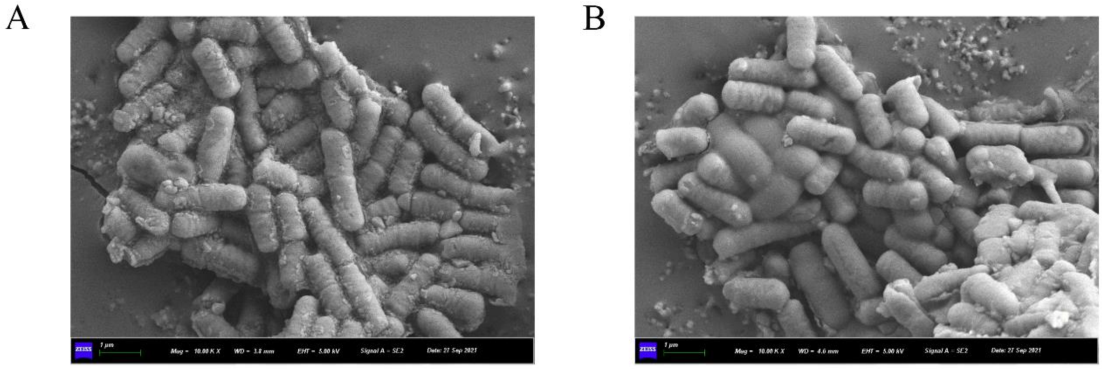

Fig. S2 Scanning electronic microscope (SEM) images of parental strain A72 (A) and evolved strain IMD4001 (B). Cells were incubated in fermentation medium containing

50 g/L glucose, and cultured at 37 °C for 24 hours. Cells were observed at 5 kV and 10, 000 magnifications, scale bars correspond to 1 μm.
