# Supplementary material for: Improvement of macrolactins production by the genetic adaptation of Bacillus siamensis A72 to saline stress via adaptive laboratory evolution
Source: Microb Cell Fact. 2022 Jul 19;21:147. doi: 10.1186/s12934-022-01871-9 (PMC9294813; doi:10.1186/s12934-022-01871-9)
Supplement: Supplementary file 2 — Additional file 2: Table S1. Oligonucleotide primers used in this study. Table S2. Plasmids and strains used in this study. 16S rRNA gene sequencing results. [file 12934_2022_1871_MOESM2_ESM.pdf]

Additional file 2

1 Additional Tables

Table S1 Oligonucleotide primers used in this study

Primer name	Sequence5`-3`	Function
pHT3101 F	GGATCCTCTAGAGTCGACCT	plasmid backbone amplification
pHT3101 R	AGCTCGAATTCGTAATCATG	
hisD F	TTACGAATTCGAGCTGATGAC GCTTCATCAAGCGC	Amplifying *hisD* cassette for homologous connection with plasmid backbone
hisD R	GACTCTAGAGGATCCAGTCGT CTTGCAGGATCTGG	
P1	CTCATTTGTTGTTCTGGCGC	Used for verifying *hisD*/*hisD*^{D41Y} overexpressing plasmids
P2	CGTTGTAAAACGACGGCCAG	
27 F	AGAGTTTGATCCTGGCTCAG	Used for verifying 16S rDNA gene
1492 R	GGTTACCTTGTTACGACTT	

Table S2 Plasmids and strains used in this study

Plasmids or strains	Description	Reference
**Plasmids**		
pHT3101	Erm^R, replicon in *B. thunngiensis*	[1]
pHT3101-WT	Erm^R, replicon in *B. thunngiensis*, harbor *hisD* cassette	This study
pHT3101-D41Y	Erm^R, replicon in *B. thunngiensis*, harbor *hisD* ^{D41Y} cassette	This study
**Strains**		
A72	Host strain	[2]
A72-WT	Over expressing wild-type *hisD*	This study
A72-D41Y	Over expressing *hisD* ^{D41Y}	This study

References

1. Lereclus D, Arantes O, Chaufaux J, Lecadet M. Transformation and expression of a cloned delta-endotoxin gene in *Bacillus thuringiensis*. FEMS Microbiol Lett. 1989;51:211-217.
2. Yi X, Gan Y, Jiang L, Yu L, Liu Y, Gao C. Rapid improvement in the macrolactins production of *Bacillus* sp. combining atmospheric room temperature plasma with

the specific growth rate index. J Biosci Bioeng. 2020;130:48-53.

2 16S rDNA sequencing results

The parental strain A72

ACATGCAGTCGAGCGGACAGATGGGAGCTTGCTCCCTGATGTTAGCGGCG
GACGGGTGAGTAACACGTGGGTAACCTGCCTGTAAGACTGGGATAACTCC
GGGAAACCGGGGCTAATACCGGATGGTTGTCTGAACCGCATGGTTCAGAC
ATAAAAGGTGGCTTCGGCTACCACTTACAGATGGACCCGCGGCGCATTAGC
TAGTTGGTGAGGTAACGGCTCACCAAGGCGACGATGCGTAGCCGACCTGA
GAGGGTGATCGGCCACACTGGGACTGAGACACGGCCCAGACTCCTACGGG
AGGCAGCAGTAGGGAATCTTCCGCAATGGACGAAAGTCTGACGGAGCAAC
GCCGCGTGAGTGATGAAGGTTTTCGGATCGTAAAGCTCTGTTGTTAGGGAA
GAACAAGTGCCGTTCAAATAGGGCGGCACCTTGACGGTACCTAACCAGAA
AGCCACGGCTAACTACGTGCCAGCAGCCGCGGTAATACGTAGGTGGCAAG
CGTTGTCCGGAATTATTGGGCGTAAAGGGCTCGCAGGCGGTTTCTTAAGTC
TGATGTGAAAGCCCCCGGCTCAACCGGGGAGGGTCATTGGAAACTGGGGA
ACTTGAGTGCAGAAGAGGAGAGTGGAATTCCACGTGTAGCGGTGAAATGC
GTAGAGATGTGGAGGAACACCAGTGGCGAAGGCGACTCTCTGGTCTGTAA
CTGACGCTGAGGAGCGAAAGCGTGGGGAGCGAACAGGATTAGATACCCTG
GTAGTCCACGCCGTAAACGATGAGTGCTAAGTGTTAGGGGGTTTCCGCCCC
TTAGTGCTGCAGCTAACGCATTAAGCACTCCGCCTGGGGAGTACGGTCGCA
AGACTGAAACTCAAAGGAATTGACGGGGGCCCGCACAAGCGGTGGAGCA
TGTGGTTTAATTCGAAGCAACGCGAAGAACCTTACCAGGTCTTGACATCCT
CTGACAATCCTAGAGATAGGACGTCCCCTTCGGGGGCAGAGTGACAGGTG
GTGCATGGTTGTCGTCAGCTCGTGTCGTGAGATGTTGGGTTAAGTCCCGCA
ACGAGCGCAACCCTTGATCTTAGTTGCCAGCATTCAGTTGGGCACTCTAAG
GTGACTGCCGGTGACAAACCGGAGGAAGGTGGGGATGACGTCAAATCATC
ATGCCCCTTATGACCTGGGCTACACACGTGCTACAATGGACAGAACAAAGG
GCAGCGAAACCGCGAGGTTAAGCCAATCCCACAAATCTGTTCTCAGTTCGG
ATCGCAGTCTGCAACTCGACTGCGTGAAGCTGGAATCGCTAGTAATCGCGG
ATCAGCATGCCGCGGTGAATACGTTCCCGGGCCTTGTACACACCGCCCGTC
ACACCACGAGAGTTTGTAACACCCGAAGTCGGTGAGGTAACCTTTATGGA
GCCAGCCGCC

The evolved strain IMD4001

AGGAAAGGTGGCGGCGTGCTAATACATGCAAGTCGAGCGGACAGATGGGA
GCTTGCTCCCTGATGTTAGCGGCGGACGGGTGAGTAACACGTGGGTAACCT
GCCTGTAAGACTGGGATAACTCCGGGAAACCGGGGCTAATACCGGATGGTT
GTCTGAACCGCATGGTTCAGACATAAAAGGTGGCTTCGGCTACCACTTACA
GATGGACCCGCGGCGCATTAGCTAGTTGGTGAGGTAACGGCTCACCAAGG
CGACGATGCGTAGCCGACCTGAGAGGGTGATCGGCCACACTGGGACTGAG
ACACGGCCCAGACTCCTACGGGAGGCAGCAGTAGGGAATCTTCCGCAATG
GACGAAAGTCTGACGGAGCAACGCCGCGTGAGTGATGAAGGTTTTCGGAT

CGTAAAGCTCTGTTGTTAGGGAAGAACAAGTGCCGTTCAAATAGGGCGGC
ACCTTGACGGTACCTAACCAGAAAGCCACGGCTAACTACGTGCCAGCAGC
CGCGGTAATACGTAGGTGGCAAGCGTTGTCCGGAATTATTGGGCGTAAAGG
GCTCGCAGGCGGTTTCTTAAGTCTGATGTGAAAGCCCCCGGCTCAACCGGG
GAGGGTCATTGGAAACTGGGGAACTTGAGTGCAGAAGAGGAGAGTGGAA
TTCCACGTGTAGCGGTGAAATGCGTAGAGATGTGGAGGAACACCAGTGGC
GAAGGCGACTCTCTGGTCTGTAACTGACGCTGAGGAGCGAAAGCGTGGGG
AGCGAACAGGATTAGATACCCTGGTAGTCCACGCCGTAAACGATGAGTGCT
AAGTGTTAGGGGGTTTCCGCCCCTTAGTGCTGCAGCTAACGCATTAAGCAC
TCCGCCTGGGGAGTACGGTCGCAAGACTGAAACTCAAAGGAATTGACGGG
GGCCCGCACAAGCGGTGGAGCATGTGGTTTAATTCGAAGCAACGCGAAGA
ACCTTACCAGGTCTTGACATCCTCTGACAATCCTAGAGATAGGACGTCCCCT
TCGGGGGCAGAGTGACAGGTGGTGCATGGTTGTCGTCAGCTCGTGTCGTG
AGATGTTGGGTTAAGTCCCGCAACGAGCGCAACCCTTGATCTTAGTTGCCA
GCATTCAGTTGGGCACTCTAAGGTGACTGCCGGTGACAAACCGGAGGAAG
GTGGGGATGACGTCAAATCATCATGCCCCTTATGACCTGGGCTACACACGT
GCTACAATGGACAGAACAAAGGGCAGCGAAACCGCGAGGTTAAGCCAATC
CCACAAATCTGTTCTCAGTTCGGATCGCAGTCTGCAACTCGACTGCGTGAA
GCTGGAATCGCTAGTAATCGCGGATCAGCATGCCGCGGTGAATACGTTCCC
GGGCCTTGTACACACCGCCCGTCACACCACGAGAGTTTGTAACACCCGAA
GTCGGTGAGGTAACCTTTATGGAGCCAGCCGCCGAAGGTGACAGAGATT
